# Experiments and Observations on the Gastric Juice and the Physiology of Digestion

**Published:** 1838-07

**Authors:** 


### Art. XIII.

*Experiments and Observations on the Gastric Juice and the Physiology of Digestion.* By William Beaumont, m.d. Surgeon in the United States' Army. *Reprinted from the Plattsburgh Edition, with Notes, by* Andrew Combe, m.d. &c.—*Edinburgh*, 1838. 8vo. pp. 319.

We are very happy to welcome to this country a work which we cannot but regard as one of the most important practical contributions to the science of physiology which the growing attention to its pursuit has of late years elicited; important, not only in the bearing of the facts detailed in it upon the theoretical explanation of one of the most universal but least understood functions of the animal system, but also in the immediate applicability of the inferences directly deducible from them to the preservation of the human body in health, and its relief from some of the most distressing maladies to which it is subject. Although many of the most valuable of these inferences were embodied in Dr. Combe's excellent work on Digestion and Dietetics, yet we were still desirous that Dr. Beaumont's own experiments and observations should be laid before the English public; and, though we regret the delay which has occurred, we are not sorry that it has been the cause of their being introduced by an editor so judicious as Dr. Combe has shown himself to be.

Many of our readers are probably aware of the nature of the case which afforded the opportunity for the enquiries, the results of which we shall briefly sketch. Alexis St. Martin was a Canadian, of French descent, of good constitution, robust and healthy; and was in the service of the American Fur Company, as a *voyageur.* On the 6th of June, 1822, being then about eighteen years of age, he was accidentally wounded by the discharge of a musket; the contents of which, consisting of powder and duck-shot, he received in his left side, being at a distance of not more than a yard from the muzzle of the gun. The charge entered posteriorly, and in an oblique direction, forward and inward, literally blowing off integuments and muscles of the size of a man's hand, fracturing and carrying away the anterior half of the sixth rib, fracturing the fifth, lacerating the lower portion of the left lobe of the lungs, the diaphragm, and perforating the stomach. The whole mass of materials forced from the musket, together with fragments of clothing and pieces of fractured ribs were driven into the muscles and cavity of the chest. When first seen by Dr. Beaumont, about half an hour after the accident, a portion of the lung, as large as a turkey's egg, was found protruding through the external wound, lacerated and burnt; and immediately below this, another protrusion, which, on further examination, proved to be a portion of the stomach, lacerated through all its coats, and pouring out the food he had taken for his breakfast, through an orifice large enough to admit the fore-finger.

That the sufferer should ultimately recover from an injury of such severe character is not the least extraordinary part of the affair. Extensive sloughing of the integuments took place within a few days, very much enlarging the original wound; and a considerable portion of the lung was also thrown off. Large portions of the ribs also exfoliated; and after some months the cartilages of the five false ribs, and the ensiform cartilage of the sternum, came away in the matter of an abscess. In about a year from the time of the accident, the injured parts were all sound and firmly cicatriced, with the exception of an aperture in the stomach and side; this was about two inches and a half in circumference, and the food and drinks constantly exuded, unless prevented by a tent, compress, and bandage. St. Martin had so far recovered as to be able to walk about and do light work, enjoying his usual good appetite and digestion, and was rapidly regaining his health and strength. Some months afterwards, a small fold or doubling of the coats of the stomach appeared forming at the superior margin of the orifice, slightly protruding, and increasing till it filled the aperture, so as to supersede the necessity for the compress and bandage for retaining the contents of the stomach. This valvular formation adapted itself to the accidental orifice, so as completely to prevent the efflux of the gastric contents when the stomach was full, but was easily depressed with the finger. The only pain experienced by St. Martin arises from a deficiency of cuticle on a space about a line wide where the skin and mucous membrane approach each other; here the cutis and nervous papillæ are unprotected, and as sensible and irritable as a blistered surface abraded of the cuticle. He has subsequently married, and become the father of a family; he is active athletic, and vigorous, taking food, drink, and exercise like other people, and enjoying almost uninterrupted good health.

It is in these latter respects that the subject of the present case differs from others in whom similar openings into the stomach have existed. Thus, M. Hallé mentions an instance in which a malady existed which permitted the interior of the stomach to be seen; and the only observation that we find respecting it is, that at each entrance of the food into the cavity, the inner membranes of the œsophagus were partially everted, so as to form a prominent circular fold at the cardia.* Other cases have occurred in which the cavity of the stomach was laid open by external wounds; but in all of them death took place in a longer or shorter time, without affording any opportunity of observing the phenomena of really *healthy* digestion. It is fortunate for the interests of science, that the remarkable opportunities presented by St. Martin's case were offered to one so able and willing to avail himself of them as Dr. Beaumont has shown himself. For nearly eleven years after his accident, St. M. was kept under Dr. B.'s observation; during a large part of the time residing in his house as a servant, for the express purpose of being experimented on. Having at two intervals returned to Canada, he was again brought by Dr. B. under his cognizance; being on one of these occasions transported, with his wife and two children, a distance of nearly two thousand miles.† For the following judicious observations on the general character of Dr. Beaumont's investigations and conclusions, we gladly acknowledge ourselves indebted to Dr. Combe's preface.

"That Dr. Beaumont eagerly and zealously availed himself of his unusual advantages, the following pages furnish ample evidence; and it would, I think, be difficult to point out any observer who excels him in devotion to truth, and freedom from the trammels of theory or prejudice. Among the disciplined physiologists of Europe, a more systematic experimenter might certainly have been found, but in Dr. Beaumont's instance, the absence of systematized enquiry—made too generally in support of a preconceived theory, and therefore apt to mislead as well as to instruct—is more than compensated by the implicit reliance which one feels can be placed on the accuracy and candour of his statements. Having no theory to support, and no favorite point to establish, Dr. Beaumont tells plainly what he saw, and leaves every one to draw his own inferences, or where he lays down conclusions, he does so with a degree of modesty and fairness of which few perhaps in his circumstances would have been capable. But, it may be said, singularly favorable as Dr. Beaumont's opportunities were, he has made no original discovery in the physiology of digestion. To a certain extent this is true; for, in the proper sense of the word, he has not made and does not claim to have made any discovery, but he has done what is at least equally essential for practical purposes. By separating the truth clearly and unequivocally from the numerous errors of fact and opinion with which it was mixed up, and thus converting into certainties points of doctrine in regard to which positive proofs were previously inaccessible, he has given to what was doubtful or imperfectly known, a fixed and positive value, which it never had before, and which, being once obtained, goes far to furnish us with a clear, connected, and consistent view of the general process and laws of diges-

* Mayo's Physiology, 4th Ed. p. 115.

† "In proof of Dr. Beaumont's disinterestedness in conducting the enquiry, (says Dr. Combe,) I may mention that I have learned from private sources that the expenses attending the various series of experiments exceeded in amount 700*l.* sterling; the whole of which was defrayed by himself, and for repayment of which he was advised to apply to Congress, on the ground of the public being interested in the promotion of scientific discovery; but, although the American Treasury was at the time literally overflowing, the application was refused."

tion. Other physiologists have attempted to effect the same end by experiments performed upon the lower animals, but these are open to so many forcible objections, that we cannot always adopt their conclusions, even where they seem to be most clearly deduced. Not to mention the cruelty inseparable from the performance of such experiments, the pain which the animal suffers necessarily disturbs the regularity of the function under examination, and in a greater or less degree vitiates the results. And even if this were not the case, the difference between the digestive organs in man and in the lower animals is so great, that it would often be unsafe to assume conclusions as applicable to the former which have been verified only in the latter." (pp. vi.-viii.)

The first portion of Dr. Beaumont's work, entitled Preliminary Observations, contains a general view of the function of Digestion, in which are embodied several of the most interesting of the observations made by the author, with the practical and theoretical inferences to which they have led him. In the second division are found the Experiments and Observations themselves, which are unfortunately too deficient in systematized arrangement to enable us to offer to our readers their general results in a condensed form. We quite agree with Dr. Combe, however, in thinking that, "though this defect diminishes the facility of access to the results, it by no means detracts from their intrinsic value. On the contrary, the very absence of systematized arrangement leaves a character of even greater trustworthiness attached to the individual observations, than if the latter had been made under the influence of some prominent guiding principle, which might have given a bias to the mind." We must refer our readers, therefore, to the work itself, the perusal of which will amply repay them, for the details; and content ourselves with laying before them some of the observations and inferences which we deem most scientifically or practically important.

The following is the account given by Dr. B. of the appearance of the villous coat of the stomach in the living state, and of its functional changes:

"The inner coat of the stomach, in its natural and healthy state, is of a light or pale pink colour, varying in its hues, according to its full or empty state. It is of a soft or velvet-like appearance, and is constantly covered with a very thin, transparent, viscid mucus, lining the whole interior of the organ. Immediately beneath the mucous coat, and apparently incorporated with the villous membrane, appear small, spheroidal, or oval-shaped, glandular bodies, from which the mucous fluid appears to be secreted.

"By applying aliment, or other irritants, to the internal coat of the stomach, and observing the effect through a magnifying glass, innumerable minute lucid points, and very fine nervous or vascular papillæ, can be seen arising from the villous membrane, and protruding through the mucous coat, from which distils a pure, limpid, colourless, slightly viscid fluid. This *fluid*, thus excited, is invariably distinctly acid. The *mucus* of the stomach is less fluid, more viscid or albuminous, semi-opaque, sometimes a little saltish, and does not possess the slightest character of acidity. On applying the tongue to the mucous coat of the stomach, in its empty, unirritated state, no acid taste can be perceived. When food or other irritants have been applied to the villous membrane, and the gastric papillæ excited, the acid taste is immediately perceptible. These papillæ, I am convinced from observation, form a part of what are called by authors the villi of the stomach. Other vessels, perhaps absorbing as well as secretory, compose the remainder. That some portion of the villi forms the excretory ducts of the vessels, or glands, I have not the least doubt, from innumerable ocular examinations of the process of secretion of gastric juice. The invariable effect of applying aliment to the internal, but exposed, part of the gastric membrane, when in a healthy condition, has been the exudation of the solvent fluid from the above-mentioned papillæ.—Though the *apertures* of these vessels could not be seen, even with the assistance of the best microscopes that could be obtained, yet the points from which the fluid issued were clearly indicated by the gradual appearance of innumerable very fine lucid specks, rising through the transparent mucous coat, and seeming to burst, and discharge themselves upon the very points of the papillæ, diffusing a limpid, thin fluid over the whole interior gastric surface: this appearance is conspicuous only during alimentation or chymification. These lucid points, I have no doubt, are the termination of the excretory ducts of the gastric vessels or glands, though the closest and most accurate observation may never be able to discern their distinct apertures. . . .

"The gastric juice never appears to be accumulated in the cavity of the stomach while fasting; and is seldom, if ever, discharged from its proper secerning vessels, except when excited by the natural stimulus of aliment, mechanical irritation of tubes, or other excitants. When aliment is received, the juice is given out in exact proportion to its requirements for solution, except when more food has been taken than is necessary for the wants of the system." (pp. 94-96.)

The interesting character of the following observations induces us to quote them in full, notwithstanding their length.

"In disease, or partial derangement of the healthy function, this membrane presents various and essentially different appearances. In febrile diathesis, or predisposition, from whatever cause,—obstructed perspiration, undue excitement by stimulating liquors, overloading the stomach with food, fear, anger, or whatever depresses or disturbs the nervous system,—the villous coat becomes sometimes red and dry, at other times pale and moist, and loses its smooth and healthy appearance; the secretions become vitiated, greatly diminished, or entirely suppressed; the mucous coat scarcely perceptible; the follicles flat and flaccid, with secretions insufficient to protect the vascular and nervous papillæ from irritation.

"There are sometime found, on the internal coat of the stomach, eruptions or deep red pimples, not numerous, but distributed here and there upon the villous membrane, rising above the surface of the mucous coat. These are at first sharp-pointed and red, but frequently become filled with white purulent matter. At other times, irregular, circumscribed red patches, varying in size or extent from half an inch to an inch and a half in circumference, are found on the internal coat. These appear to be the effect of congestion in the minute blood-vessels of the stomach. There are also seen at times small aphthous crusts in connexion with these red patches. Abrasion of the lining membrane, like the rolling up of the mucous coat into small shreds or strings, leaving the papillæ bare for an indefinite space, is not an uncommon appearance. These diseased appearances, when very slight, do not always effect essentially the gastric apparatus. When considerable, and particularly when there are corresponding symptoms of disease,—as dryness of the mouth, thirst, accelerated pulse, &c.—*no gastric juice can be extracted, not even on the application of alimentary stimulus.* Drinks received are immediately absorbed or otherwise disposed of, none remaining in the stomach ten minutes after being swallowed. Food taken in this condition of the stomach remains undigested for twenty-four or forty-eight hours, or more, increasing the derangement of the whole alimentary canal, and aggravating the general symptoms of disease. After excessive eating or drinking, chymification is retarded; and, although the appetite be not always impaired at first, the fluids become acrid and sharp, excoriating the edges of the aperture, and almost invariably produce aphthous patches, and the other indications of a diseased state of the internal membrane mentioned above. Vitiated bile is also found in the stomach under these circumstances, and flocculi of mucus are much more abundant than in health. Whenever this morbid condition of the stomach occurs, with the usual accompanying symptoms of disease, there is generally a corresponding appearance of the tongue. When a healthy state of the stomach is restored, the tongue invariably becomes clean." (pp. 98-100.)

The dietetic principles founded upon these observations, and applicable both to the maintenance of health and the treatment of disease, are too obvious to require elucidation; but Dr. Combe's commentary is so apposite that we cannot refrain from adding it.

"Many persons, who obviously live too freely, protest against the fact, because they feel no immediate inconvenience, either from the quantity of food or the stimulants in which they habitually indulge; or, in other words, because they experience no pain, sickness, or headach,—nothing, perhaps, except slight fulness and oppression, which soon go off. Observation, extended over a sufficient length of time, shows, however, that the conclusion drawn is entirely fallacious, and that the real amount of injury is not felt at the moment, merely because, for a wise purpose, nature has deprived us of any consciousness of either the existence or the state of the stomach during health. In accordance with this, Dr. Beaumont's experiments [observations] prove that extensive erythematic inflammation of the mucous coat of the stomach was of frequent occurrence in St. Martin after excesses in eating, and especially in drinking, even when no marked general symptom was present to indicate its existence. Occasionally febrile heat, nausea, headach, and thirst were complained of, but not always. Had St. Martin's stomach, and its inflamed patches, not been visible to the eye, he too might have pleaded that his temporary excesses did him no harm; but, when they presented themselves in such legible characters that Dr. Beaumont could not miss seeing them, argument and supposition were at an end, and the broad fact could not be denied." (p. 317.)

The examination of the characters of pure gastric juice was, of course, not omitted. This fluid was obtained by the introduction of an elastic tube into the stomach; the partial contact of which with its parietes excited the secretion from the points it stimulated. As these points bear but a small relation to the whole internal surface of the stomach, Dr. B. was never able to obtain in this manner more than one and a half or two ounces of the fluid at one time; and ten, fifteen, or more minutes were necessary to collect even this small quantity. Its extraction was generally attended by that peculiar sensation at the pit of the stomach termed sinking, with some degree of faintness, which rendered it necessary to stop the operation. The principal active ingredient contained in it appears to be muriatic acid, which exists free in sufficient quantity to produce a distinctly sour taste, and to throw down a large quantity of chloride of silver from a solution of the nitrate. From the experiments made with the fluid thus obtained upon various edible substances, Dr. Beaumont arrives at the conclusion, which appears to us unimpregnable, that chymification is essentially a chemical process, as maintained by Spallanzani; and that the solvent action of the gastric juice, aided by the *motions* of the stomach and the natural *warmth* of the system, is the essential agent in the operation. We need scarcely point out how completely this inference has been confirmed by the late experiments of Müller and Schwann upon artificial digestion; which was effected by means of a *manufactured* gastric juice.* We must say that *à priori* considerations alone would lead us to reject any other doctrine, unless supported by very strong evidence; for, as we are plainly to regard the stomach and alimentary canal but as an inversion of the external surface, contrived for adapting the function of absorption to the conditions of animal existence, there is no reason why substances contained in this cavity should become more *vitalized* than others applied to the skin, or

* See British and Foreign Med. Review, Vol. IV., p. 201; and Müller's Physiology, p. 545, &c.

than the air which is introduced into the lungs. Aliment is not really introduced into the system until the process of absorption commences; and with that process, we believe, the organization of the constituents of the fluid, and their endowment with vital properties, to commence. Dr. Beaumont found that the mixture of bile and pancreatic juices with chyme separated from it a fluid which he terms *crude chyle*, and which he regards as that taken up by the absorbents. He does not inform us, however, whether it contains globules or possesses the power of coagulating; both which are characteristics of true chyle.

The quantity of gastric juice secreted by the stomach for the solution of its contents does not depend upon the quantity of food introduced into the cavity, but upon the general requirements of the system. This is a principle of the highest importance in a practical view, and cannot be too constantly borne in mind. A definite proportion of aliment only can be perfectly digested in a given quantity of the fluid, the action of which, like other chemical operations, ceases after having been exercised on a fixed and definite amount of matter.

"When the juice becomes saturated, it refuses to dissolve more; and, if an excess of food have been taken, the residue remains in the stomach, or passes into the bowels in a crude state, and frequently becomes a source of nervous irritation, pain, and disease for a long time; or until the *vis medicatrix naturæ** restores the vessels of this viscus to their natural and healthy actions, either with or without the aid of medicine." . . . "Derangement of the digestive organs, slight febrile excitement, fright, or any sudden affection of the passions, causes material alterations in its appearance. Overburthening the stomach produces acidity and rancidity in this organ, and retards the solvent action of the gastric juice. General febrile irritation seems entirely to suspend its secretion into the gastric cavity, and renders the villous coat dry, red, and irritable. Under such circumstances it will not respond to the call of alimentary stimulus. Fear and anger check its secretion also; the latter causes an influx of bile into the stomach, which impairs its solvent properties." (p. 77.)

It would seem, then, that the connexion between the angry passions and the flow of bile, recognized by the term *choler*, is not so fabulous as some have imagined.

There cannot be a stronger argument as to the necessity of a careful regulation of the ingesta for the preservation of health than that derived from the relation between the quantity of gastric juice secreted and the requirements of the system, above alluded to. If a temporary increase in the quantity of this fluid were all the disorder occasioned by an excess in the quantity of food, the mischief would pass entirely unnoticed until the diminished power of the secreting organs prevented them from obeying the call. But we find the fact really to be, that, if a more than ordinary quantity of food be taken, and there is no demand for it in the system, part of it is left undissolved in the stomach, and thus indigestion is produced.

"But if the ingestion of a large quantity be in proportion to the calls of nature, which sometimes happens after an unusual abstinence, it is probable that more than the usual supply of gastric juice is furnished; in which case the apparent excess is in exact ratio to the requirements of the economy, and never fails to produce a sense of quiescent gratification and healthful enjoyment. A great deal depends upon habit in

* We must hint to Dr. Beaumont that this phrase is not very dissimilar to one which he quotes with disapprobation from another writer, "the foresight of the vital principle," as meaning everything or nothing.—Rev.

this respect. Our western Indians, who frequently undergo long abstinences from food, eat enormous quantities, when they can procure it, with impunity."

"There appears to be a sense of perfect intelligence conveyed from the stomach to the encephalic centre, which, in health, invariably dictates what quantity of aliment (responding to the sense of hunger, and its due satisfaction,) is naturally required for the purposes of life; and which, if noticed and properly attended to, would prove the most salutary monitor of health, and effectual preventive of, and restorative from, disease. It is not the sense of satiety; for this is beyond the point of healthful indulgence, and is nature's earliest indication of an abuse and overburthen of her powers to replenish the system. It occurs immediately previous to this, and may be known by the pleasurable sensation of perfect satisfaction, ease, and quiescence of body and mind." (p. 55.)

We have not space to enter into the interesting description given by Dr. Beaumont of the motions of the stomach: we may mention, however, that there appears to be a complete revolution of its contents in periods of from one to three minutes, during the process of digestion, and that the whole contents of the stomach are thus mixed together, without any line of separation between the new and old food, as Dr. W. Philip supposed. As to the effect of these movements, Dr. B. remarks:

"The vermicular motions, being excited by mechanical irritation, not only carry the ingestæ into all parts of the stomach, and diffuse its mechanical influence throughout the whole inner surface of this organ, but by this means they uniformly mix the aliment with the gastric juice, which is constantly being secreted in proportion to the quantity of food received into the stomach, (unless that be too much for the wants of the economy,) until chymification be completed. Some stimulus seems to be necessary to continue the motions of the stomach after chymification is accomplished, in order to effect its complete discharge into the lower bowels: and it appears highly probable that the compound fluid of gastric juice and aliment, or chyme, by its acquired acid properties, affords this stimulus, and propagates the contractile motions of this organ, even after the mechanical irritation of the crude food ceases. This fluid acquires new chemical properties, becomes more acid and stimulating, as chymification advances, until it is completed. When it is all transferred to the duodenum, the motions of the stomach cease." (p. 82.)

With a few words respecting Dr. Beaumont's theory of hunger, we must close our account of the first part of his volume. The experiments and observations contained in the second have each an independent interest, and bear upon a number of minor points, to enumerate which alone would be tedious: we must therefore refer our readers to the work itself, with the assurance that they will find much in it to instruct and interest them. After explaining his grounds for withholding assent to any of the common opinions regarding the cause of the sensation of hunger, Dr. Beaumont says,

"My impression is, that the sensation of hunger is produced by a *distension* of the gastric vessels, or that apparatus, whether vascular or glandular, which secretes the gastric juice, and is believed to be the effect of repletion by this fluid. One reason, among others, for this belief is the established fact that the internal sensations referred to different organs, as has been previously alluded to, are caused by some modified action or condition of the parts in the tissues of the organ itself. The modification in the parts to which the sense of hunger is invariably referred, I conceive to be a distension, by the gastric juice, of a particular set of vessels or glands, constituting in part the erectile tissue of the villous coat of the stomach. The sensation varies according to the different degrees or states of distension, from the simplest desire to the most painful sense of hunger; and is allayed or increased in proportion to the application or refusal of alimentary stimulus to the excretory vessels." (p. 47.)

He grounds his belief that the fluid is secreted and stored up in the vessels, on the suddenness of its appearance when called for by irritation of the mucous surface of the stomach; but, as Dr. Combe justly remarks, "when we remember the equal rapidity with which saliva flows into the mouth of a hungry man, when a good roast of meat is placed before him, we shall be disposed to question the fact; unless, indeed, we hold that the saliva also was stored up in its vessels ready for use. Besides, bad news cannot instantly empty the gastric vessels of their contents, and yet they dispel appetite most effectually." Moreover, we feel by no means inclined to agree with Dr. B.'s general proposition, that pain and uneasiness always arise from distended vessels. Dr. Combe regards the sensation of hunger as arising from "a certain condition of the stomachic nerves, arising out of their relation to the state of the general system and to the brain;" this state of the system causing the stomachic nerves to excite a feeling of hunger, in the same manner as atmospherical vibrations, impinging upon the nerves of hearing, produce the feeling of sound. We cannot altogether satisfy ourselves with this explanation; since we are convinced that there is a more direct cause of the sensation to be looked for in the condition of the stomach itself, by the fact that mere distension of the viscus with innutrient substances will temporarily relieve hunger. As to the exact nature of this condition, we must confess ourselves in the dark; but we quite accord with the remark of Dr. Alison, that, "whatever be the conditions under which the nerves of the stomach become the seat of these sensations, it is certain that, in the healthy state, they are a true index, not only to the state of the stomach, but to the immediate wants of the system at large." We are ourselves inclined to attribute the feeling to the determination of blood to the organ, which must be the preparatory step to the copious effusion of the gastric fluid; and we are by no means certain that the phenomenon of the digestion of the coats of the stomach and of the surrounding viscera after death* does not show (notwithstanding Dr. Combe's argument) that the gastric juice may be stored up in cavities of the tissue between the time of its secretion and that of its discharge. At this conclusion it seems necessary to arrive, unless we are disposed to allow that the secretion is formed after death.

There is still required a systematic and carefully conducted series of experiments, for the purpose of ascertaining the relative digestibility of different kinds of food. It is obvious that the time required for artificial solution of various aliments in gastric juice cannot be regarded as a sufficient indication; since it may be readily imagined that the consistence of one kind of solid may be of such a nature as to require the peculiar movements of the stomach for the separation of its particles, and that it may thus appear, if tested in this manner only, less digestible than substances which more readily dissolve. Moreover, it is very difficult to extract gastric juice by the methods formerly practised, with any certainty of its purity, and of its having the same powers at one time as at another. The case of St. Martin, therefore, affords us a most valuable

* This digestion is effected by the gastric juice, not only in carnivorous and in omnivorous, but in herbivorous animals. We believe it is a rare thing to find a prepared calf's stomach (such as is used for rennet) without a perforation of this kind.

opportunity for this important investigation. Dr. Beaumont's chief aim was to ascertain the nature and laws of the digestive process; and his observations upon the comparative digestibility of different substances were thus too incidental to be relied on as minutely accurate, though in a general way worthy of attention. The table, therefore, in which the results which bear upon this subject have been thrown together cannot be regarded as exhibiting certainties, but only approximations to truth. The rapidity of digestion is so much influenced by the quantity eaten, the degree of preparatory mastication, the amount of exercise, the mode of life, and state of health, that no *positive* conclusions on this point can be drawn, except where due attention has been paid to all these modifying circumstances. We have much pleasure, therefore, in doing our best to draw attention to the following suggestion of Dr. Combe's:

"In the second edition of the 'Physiology of Digestion,' I ventured to suggest that some of our scientific associations, such as the Royal Society or British Association, would do science a service and themselves an honour, by using their influence and means to have St. Martin brought over to this country, and the remainder of the subject fully investigated under the direction of a committee of their number. An opportunity of this kind may never occur again, and it will be a source of lasting regret, and even of merited reproach, if it be allowed to pass away without being turned to the best possible account. If the suggestion now thrown out shall ever be acted upon, special care should be taken not to injure St. Martin's health, by withdrawing him entirely from his accustomed diet and mode of life; otherwise, the whole value of the experiment may be lost,—the object being to ascertain the laws and conditions of HEALTHY DIGESTION." (*Pref.* p. xiv.)

We cannot but hope that, if the object be taken up by the influential members of our profession, and properly brought forwards at the ensuing meeting of the British Association, it may be effectually accomplished. We are sure that there is not an enlightened member in the higher ranks of the profession who would not gladly contribute a small subscription, for a limited number of years, to aid any of our public bodies who might, in the first instance, advance a sufficient sum from their funds to get St. Martin conveyed to this country. In the event of this laudable design being carried into effect, we know no individual to whom the task of superintending the experiments could be confided with greater confidence than to the accomplished and honorable editor of Dr. Beaumont's book.

---

